# Correction: Inferring exemplar discriminability in brain representations

**DOI:** 10.1371/journal.pone.0250474

**Published:** 2021-04-19

**Authors:** Hamed Nili, Alexander Walther, Arjen Alink, Nikolaus Kriegeskorte

The captions for Figs 1–10 are missing. Please see all complete, correct figure captions here.

**Fig 1:**
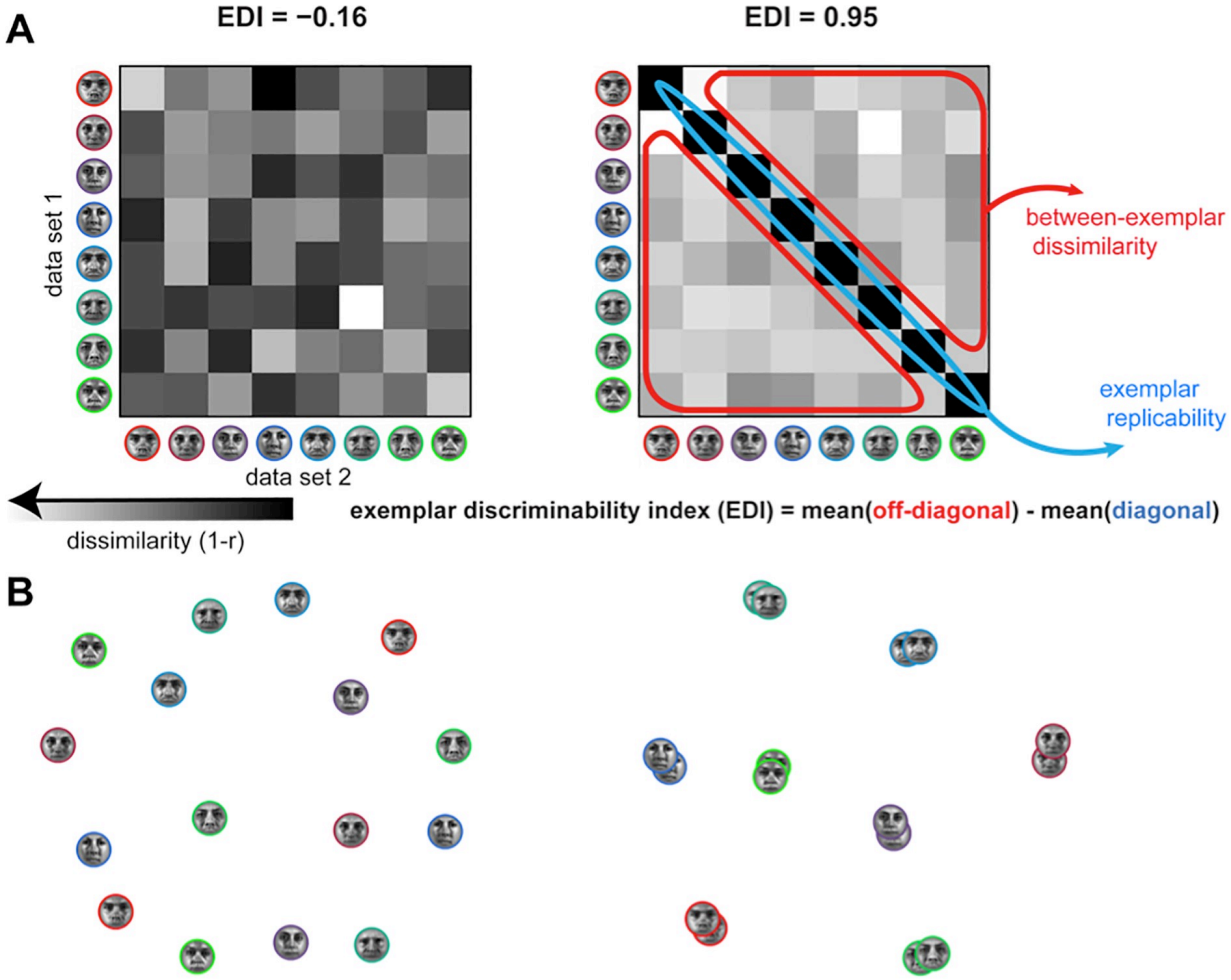
Definition of the exemplar discriminability index (EDI). **(A)** The exemplar discriminability index (EDI) is defined as the difference of the average between-exemplar and average within-exemplar distance. Split-data RDMs (sdRDMs) are shown for two scenarios. In the scenario on the left, the face exemplars are not discriminable and the EDI is insignificant (H_0_ simulation). In the scenario on the right, the EDI is large and exemplars are discriminable (H1 simulation). **(B)** Applying multi-dimensional scaling (MDS; Kruskal and Wish, 1978; Torgerson, 1958) allows simultaneous visualisation of the representational geometries from both data sets (H_0_ on the left, H1 on the right). Exemplars are colour-coded and there are two pattern estimates for each exemplar. MDS gives a low dimensional (2D here) arrangement, wherein the distances between projections approximate the original distances in the high-dimensional pattern space. Here, to obtain MDS plots, we consider a distance matrix composed of comparisons between any two patterns (aggregated from both datasets A and B). When the EDI is significantly positive (right), repetitions of the same exemplar yield patterns that are more similar to each other than presentations of two different exemplars.

**Fig 2:**
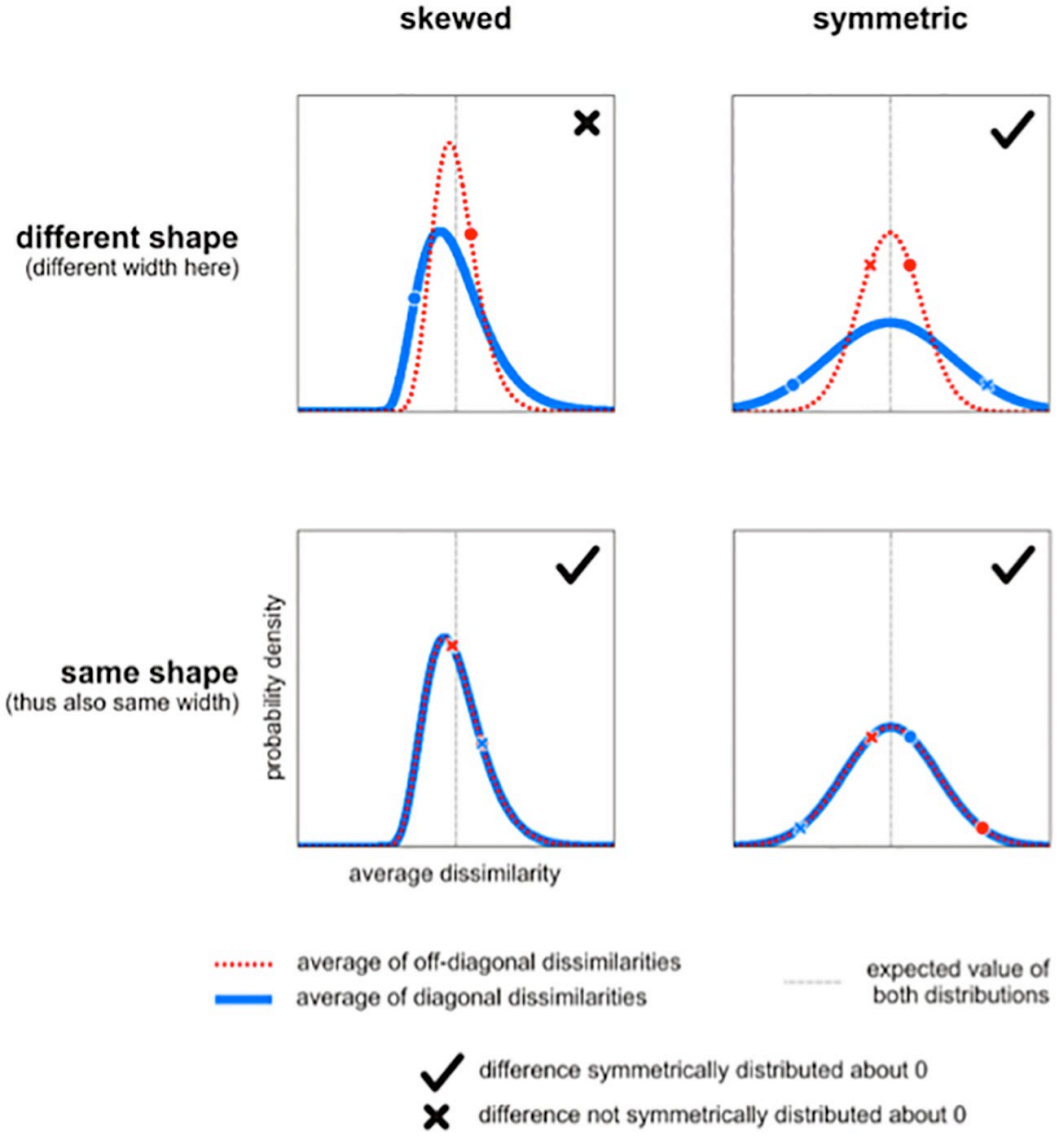
The EDI is not strictly symmetrically distributed under H_0_. EDI is the difference between the average diagonal entries (blue) and the average off-diagonal entries (red). The distributions of these averages can be skewed or non-skewed and have the same or different shapes. Note that under H_0_, the two distributions have the same expected value (gray vertical line). When the two distributions are both symmetric (right panels) or when they are both of the same shape (lower panels), their difference will be symmetrically distributed about 0. To see this, consider the fact that for any two points (one from each distribution, e.g. blue X and red X), another equally probable two points exist (blue O and red O) such that their difference has the same absolute value and the opposite sign. However, the distribution can be asymmetric (and thus non-Gaussian) when the two distributions are both skewed and have different shapes (upper left panel).

**Fig 3:**
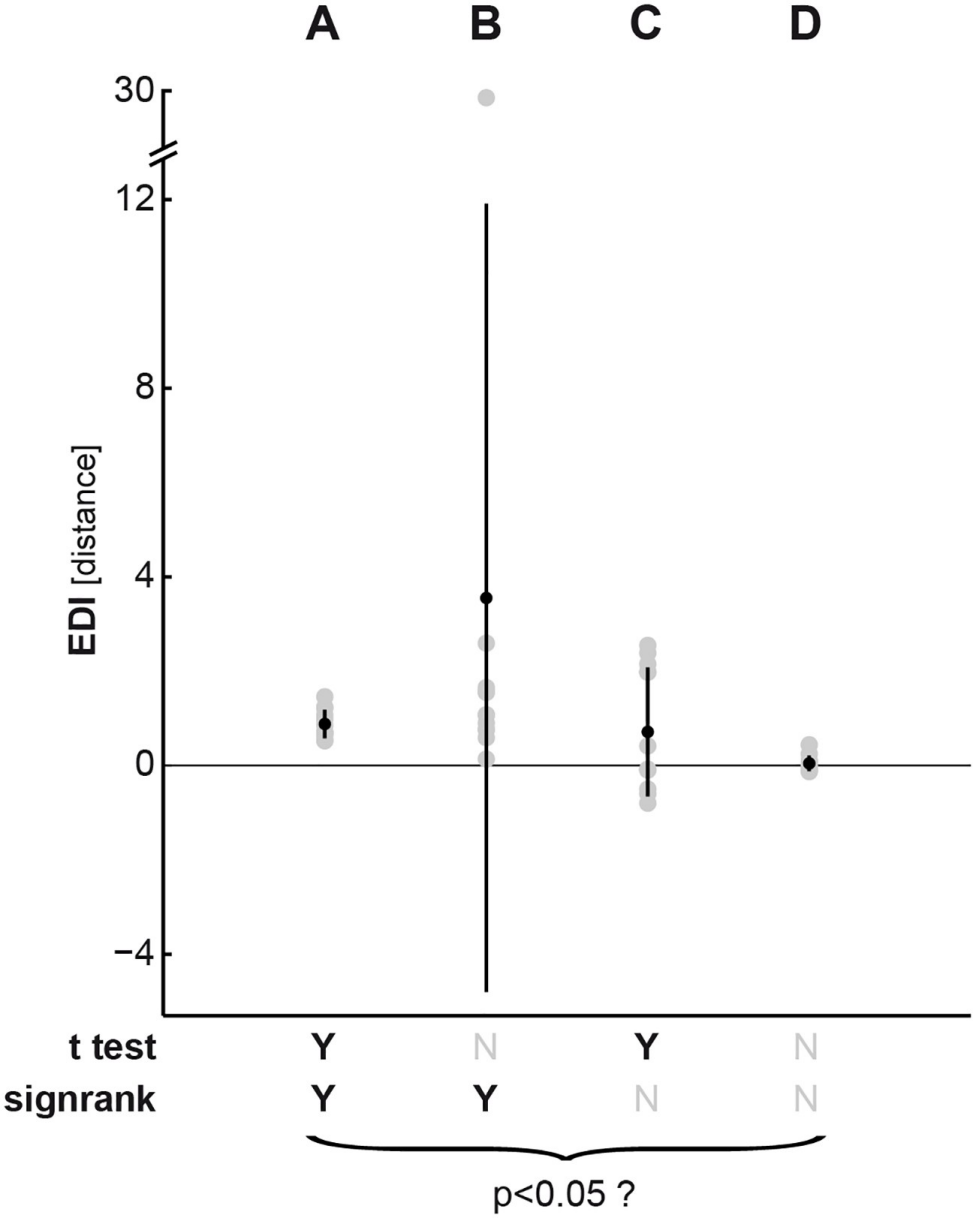
*t* test and Wilcoxon signed-rank tests examine different null hypotheses about the data distribution. The graph depicts four different sets of simulated EDIs (A to D) sampled from Gaussian distributions with different means and variances. Gray points indicate the sample data. The superimposed black point depicts the mean of each sample. The sample variance around its mean is depicted by black lines emanating from the mean (*i*.*e*., the black dot). Each set consists of 12 values, representing 12 subjects, which are submitted to both a *t* test and a Wilcoxon signed-ranked test. Both tests are right-tailed (meaning the tail extending into the positive direction) and test the null hypothesis that the data come from a distribution with zero mean (meaning no exemplar information across participants). p-values were pronounced significant (Y) if they passed the conventional threshold of p<0.05. Otherwise, they were unsignificant (N). (A) The sample mean is positive and the sample variance is small, hence both tests pass the significance threshold. (B) The sample mean is positive. However, an extreme value in the sample inflates the variance. Therefore the *t* test does not return a significant p-value anymore. (C) The mean is positive, but the sample contains a considerable number of negative EDIs. Accounting for the weight of the ranks of the data points, the Wilcoxon-sigend rank test penalizes this presence of negative data points, therefore its p-value does not pass the significance threshold. (D) The sample mean is close to zero, hence neither test indicates significant exemplar information.

**Fig 4:**
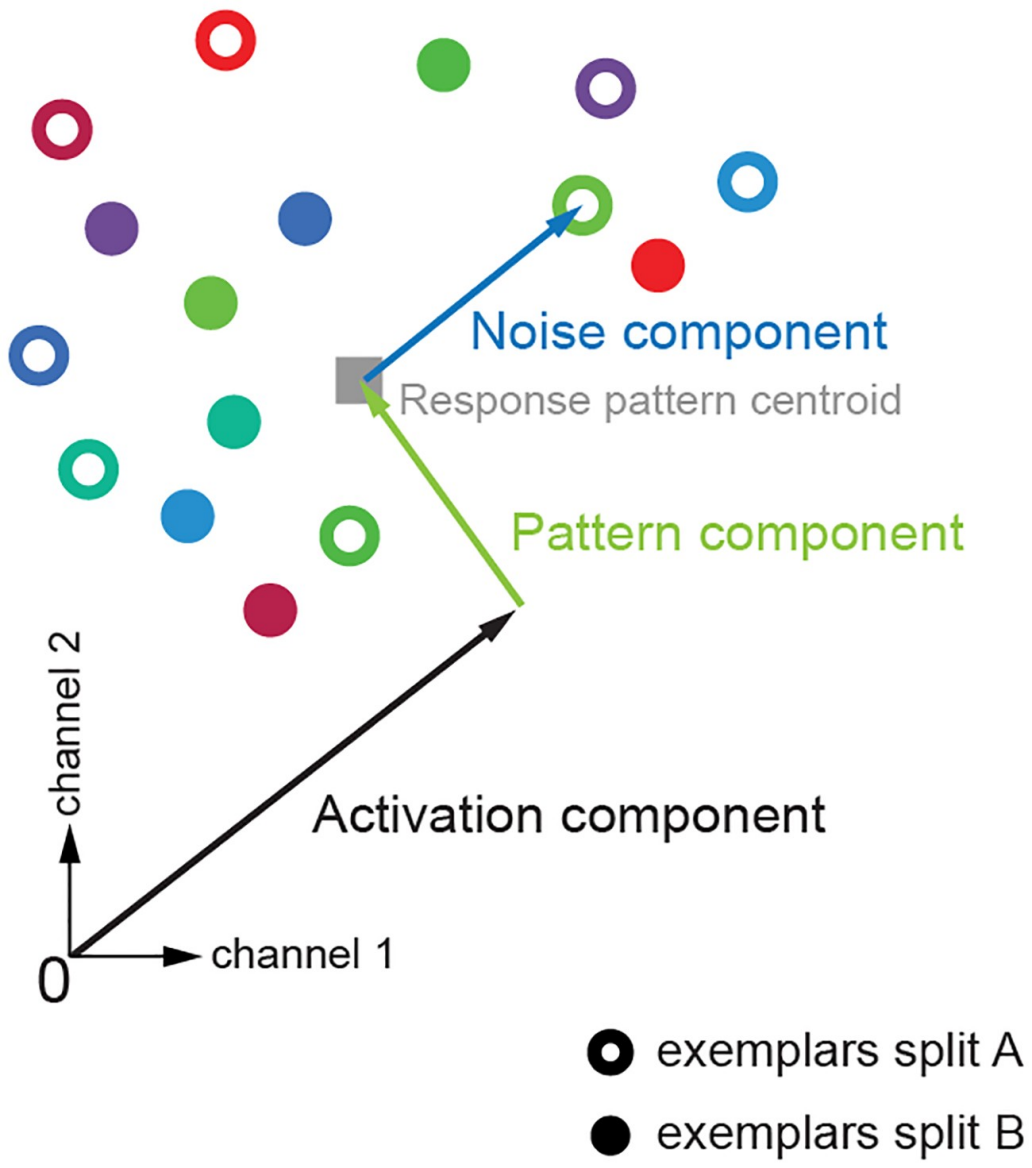
H_0_ simulation settings. We use simulations to test the distribution of EDIs under the null hypothesis, H_0_ (here illustrated for only two response channels). For both datasets 1 and 2, patterns of exemplars were simulated in a multi-dimensional response space. The number of dimensions of the response space is the same as the number of response channels (e.g. voxels in a region of interest in fMRI data). Response patterns were simulated by first moving along the all-ones vector to reach the average activation, whose strength was determined by the *activation component*. A Gaussian pattern with a *pattern component* variance was added to the *activation component*, resulting in the response pattern centroid. Individual exemplars of each data split were then generated by adding random Gaussian noise with a *noise component* variance to the centroid. At each iteration of the simulation, the same centroid underlay all exemplars in both data splits (as exemplars are indiscriminable under H_0_). Exemplar labels were randomly assigned to experimental conditions and their repetitions. For details of the simulations see section 2.4.1.

**Fig 5:**
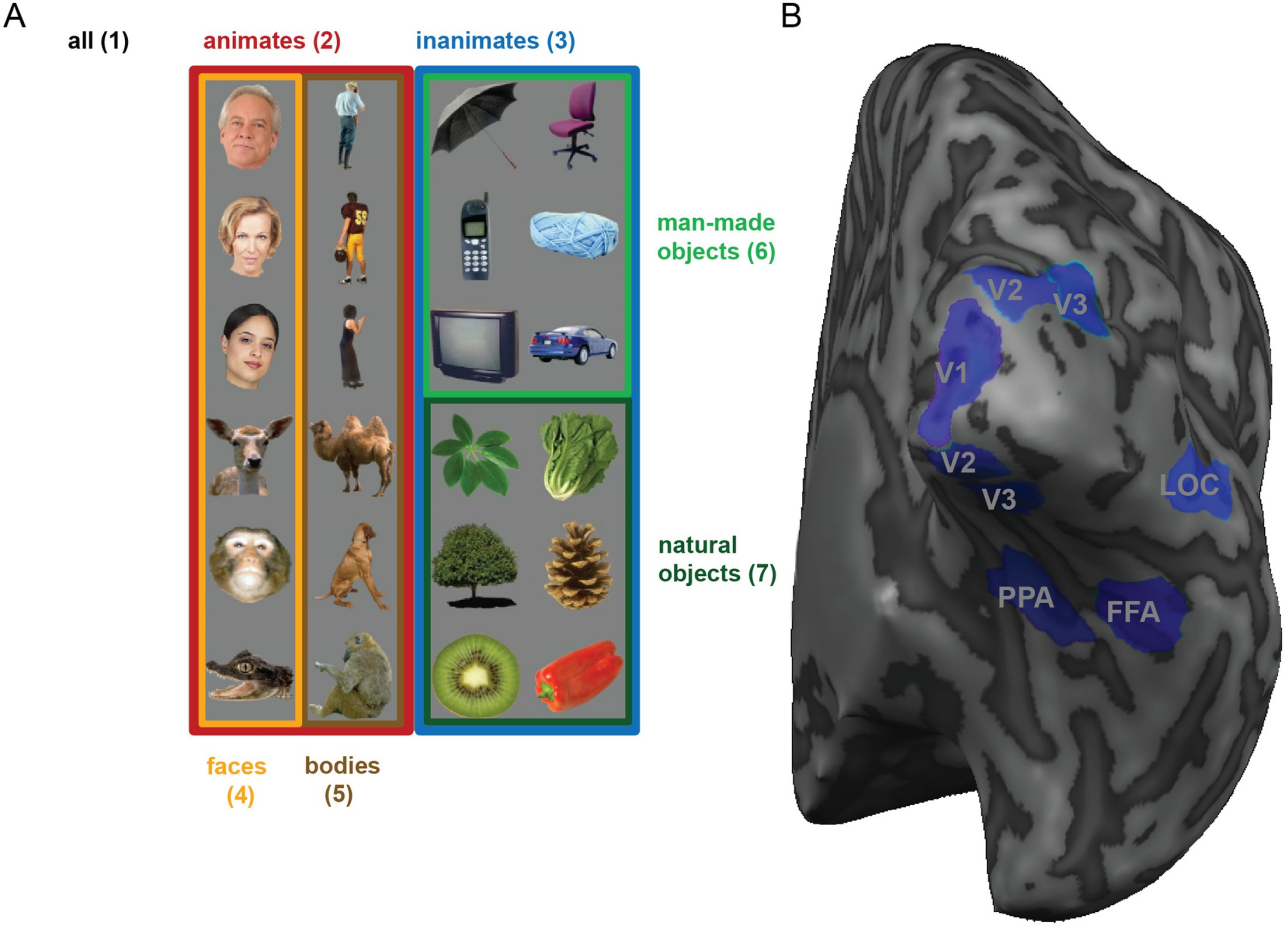
Different exemplar sets (A) and ROIs (B) considered for the analysis of real fMRI data. In order to evaluate the different tests, we apply the tests to real fMRI data. Having data from different regions of interest (V1, V2, V3, LOC, FFA and PPA, displayed for an example subject on the right) and testing the discriminability for different exemplar sets (*i*.*e*. different sets of experimental conditions, displayed on the left) confronts the tests with a wide range of situations. To obtain an estimate of the type I error, different tests were applied to randomised fMRI data (simulating the null hypothesis with real data). Seven subsets of conditions are considered: 1) all 24 images 2) 12 images belonging to the animate category 3) 12 images belonging to the inanimate category 4) 6 face exemplars 5) 6 images of bodies 6) 6 man-made objects 7) 6 natural objects.

**Fig 6:**
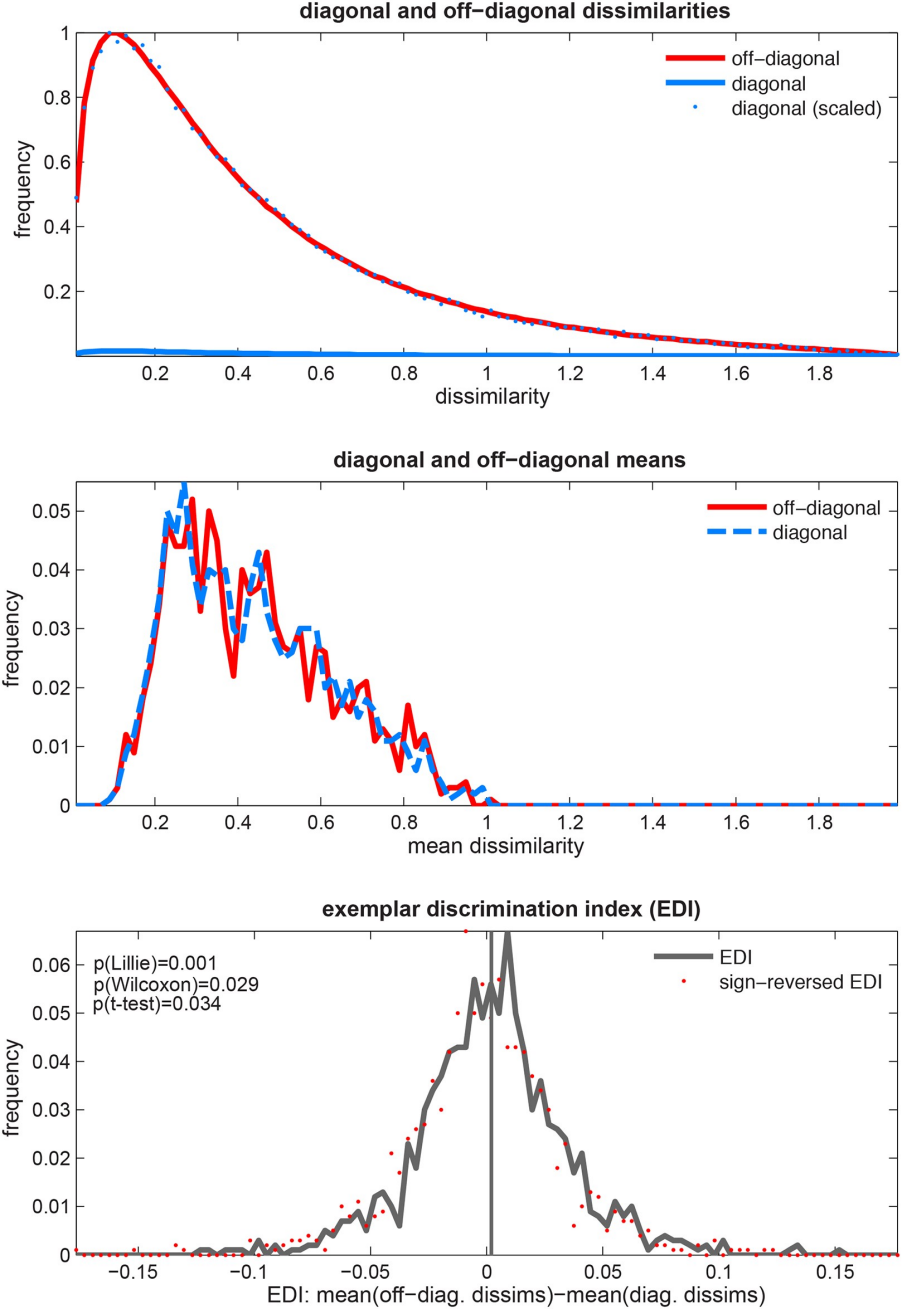
The EDI can be non-Gaussian under the null hypothesis (H_0_). *Top panel*: The distribution of the diagonal (blue) and off-diagonal (red) dissimilarities. Distributions are obtained from the dissimilarities aggregated across pairs and simulated subjects. There are fewer diagonal entries but they are sampled from the same distribution as off-diagonal dissimilarities under the null hypothesis. Therefore the two distributions differ only by a scaling factor. *Middle panel*: The distribution of the diagonal and off-diagonal average dissimilarities. Data were simulated for each subject (1,000 in total) and the distributions were obtained by pooling the averages across all subjects. *Bottom panel*: The EDI distribution in this case is significantly non-Gaussian. Furthermore, a one-sided *t* test or Wilcoxon signed rank test both yield significant p-values. This means that EDI is not strictly zero-mean and Gaussian under H_0_. This could potentially inflate both types of error and weaken inference based on the *t* test. For each of the 1,000 subjects, data were simulated for 64 exemplars and five response channels.

**Fig 7:**
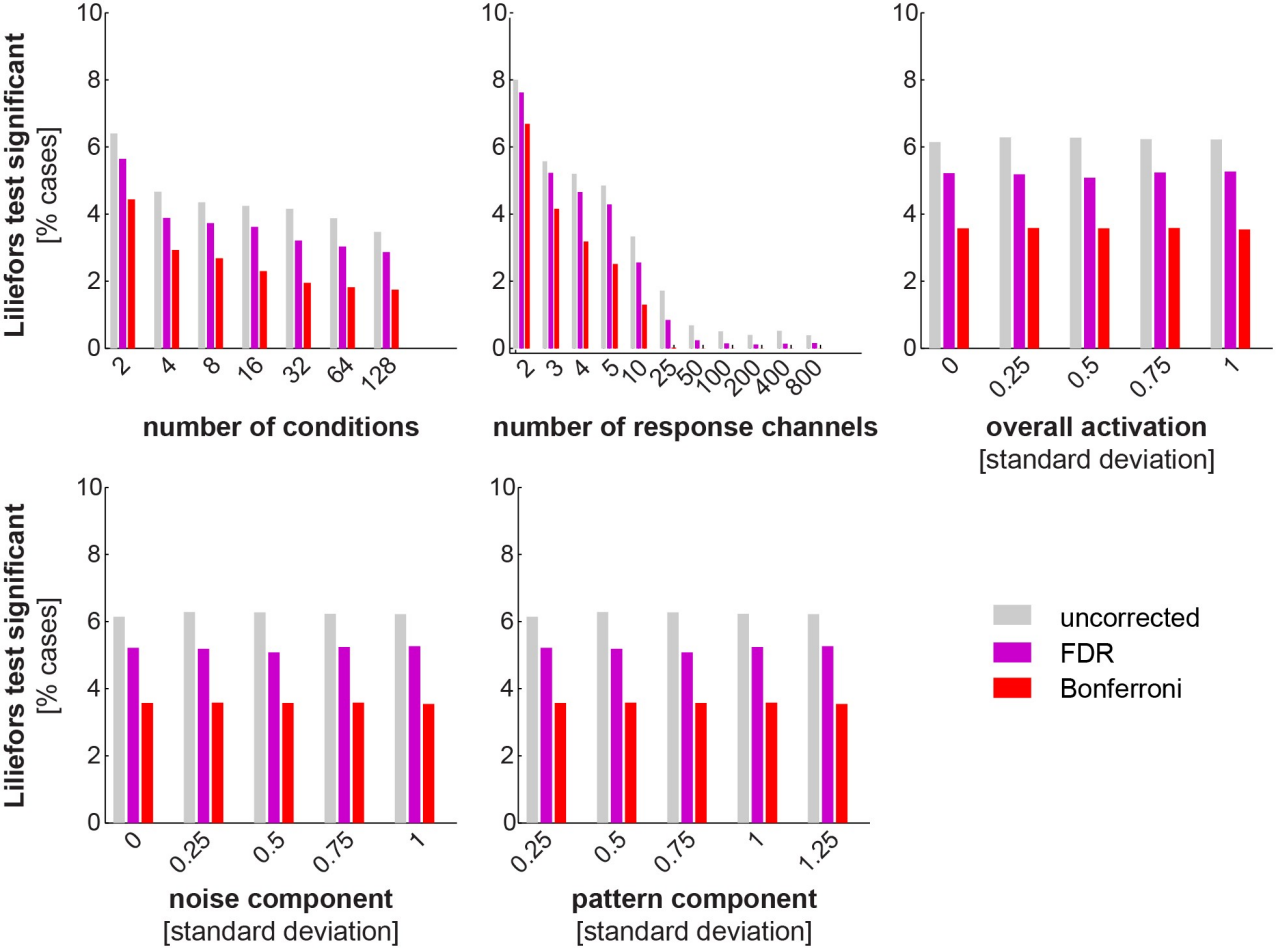
Marginal histograms of deviations from Gaussianity of the simulated EDI null distribution. In order to richly characterise the properties of the EDI distribution under H_0_, we simulate data for a large number of subjects for a range of values of any parameter. At every point of the cross-product parameter space, sdRDMs (based on Pearson correlation distance) were obtained from simulated patterns for 10,000 subjects (patterns were simulated independently for each subject). Lilliefors tests (testing whether the distribution of EDIs is significantly non-Gaussian) were applied to the EDI distributions. These bars correspond to results from the Lilliefors Gaussianity test. A significance threshold of 5% was applied to the tests. Each column also corresponds to one dimension of the parameter space. The first two (number of exemplars and number of response channels) mainly depend on the experiment and analysis and the other three characterise the multivariate response space. For example, the red bar on the leftmost bar-graph would correspond to the proportion of all Lilliefors tests that were significant (p < 0.05, Bonferroni correction) when the number of conditions were fixed at 2, 4, 8, etc. and the other parameters had any possible value. Two other tests were performed in a similar way to test EDIs for the simulated data under H_0_. One was a one-sided Wilcoxon signed rank test and the other a one-sided *t* test. The signed rank test was applied to assess whether the EDIs were zero centered and the *t* test was applied to obtain an estimate of the false-positive rates. Interestingly, for both tests less than 1% violations occurred (p < 0.05) and none of those survived correction for multiple comparisons (FDR and Bonferroni, p < 0.05).

**Fig 8:**
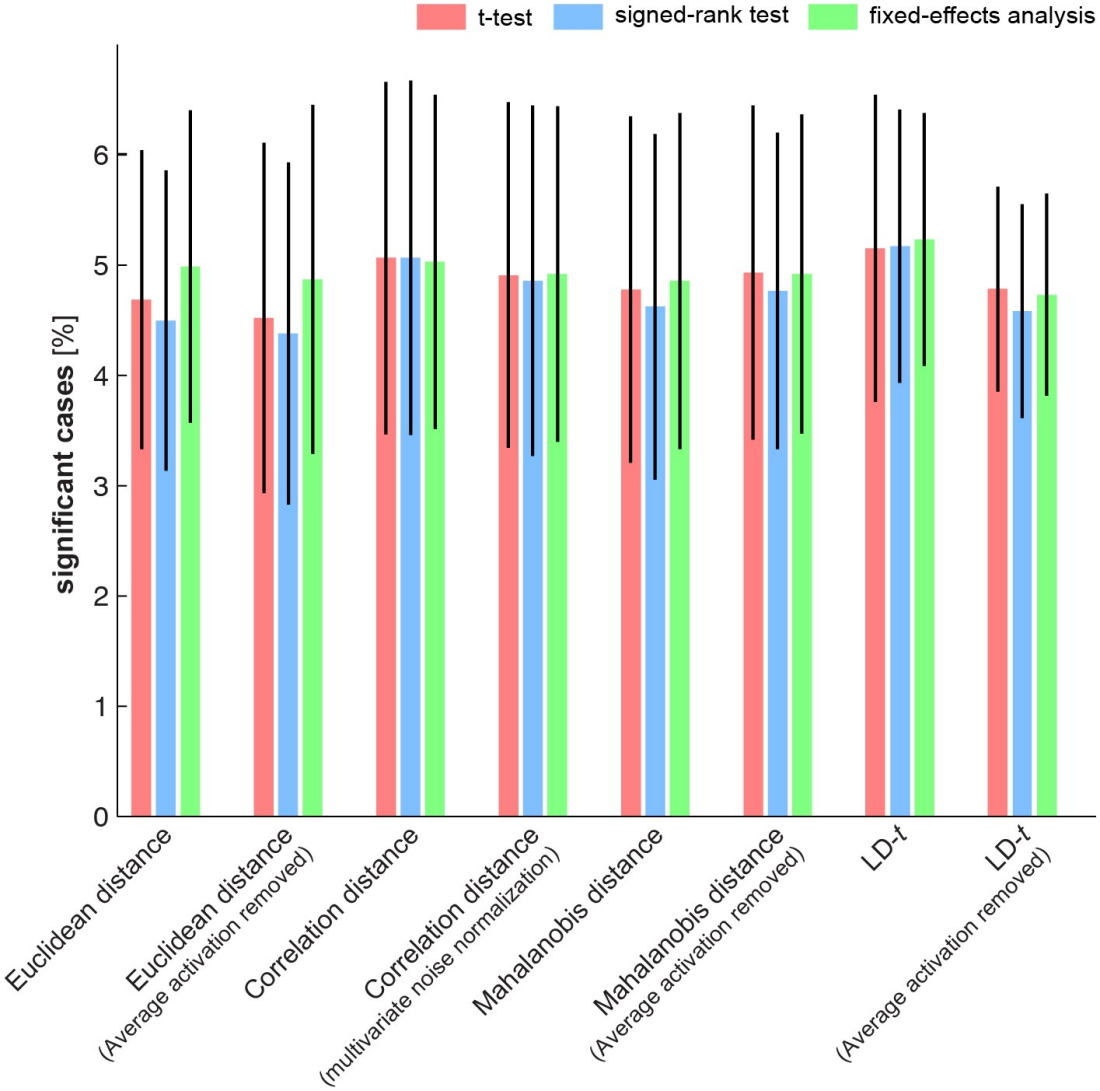
False-positives rates for different tests of exemplar discriminability. We estimated the false positive rate (type-I error rate) by applying tests to randomised fMRI data (simulating H_0_, see section 2.4.2 for details). The null data were simulated for a large number of iterations. At each iteration an ROI was randomly selected and data for each subject from that ROI were randomised by permuting the condition labels for a full set containing experimental conditions from both data splits. Randomisation was independently carried out for the two data splits. Under the null hypothesis, exchangeability implies that there should not be a pattern difference between replications of the same exemplar and a different exemplar. At each iteration (1,000 in total), we simulated group level data under H_0_ and applied all tests to it. We then computed the average percentage of false positives (proportion of significant cases from the total number of 1,000 simulated cases, *i*.*e*. height of the bars) and their standard deviation (error bars). All tests had a false positive rate that was not significantly different from 5% after a threshold of 0.05 was applied to the tests (p > 0.5, two-sided Wilcoxon test). This means that all tests protect against false positives at an expected rate and are therefore valid.

**Fig 9:**
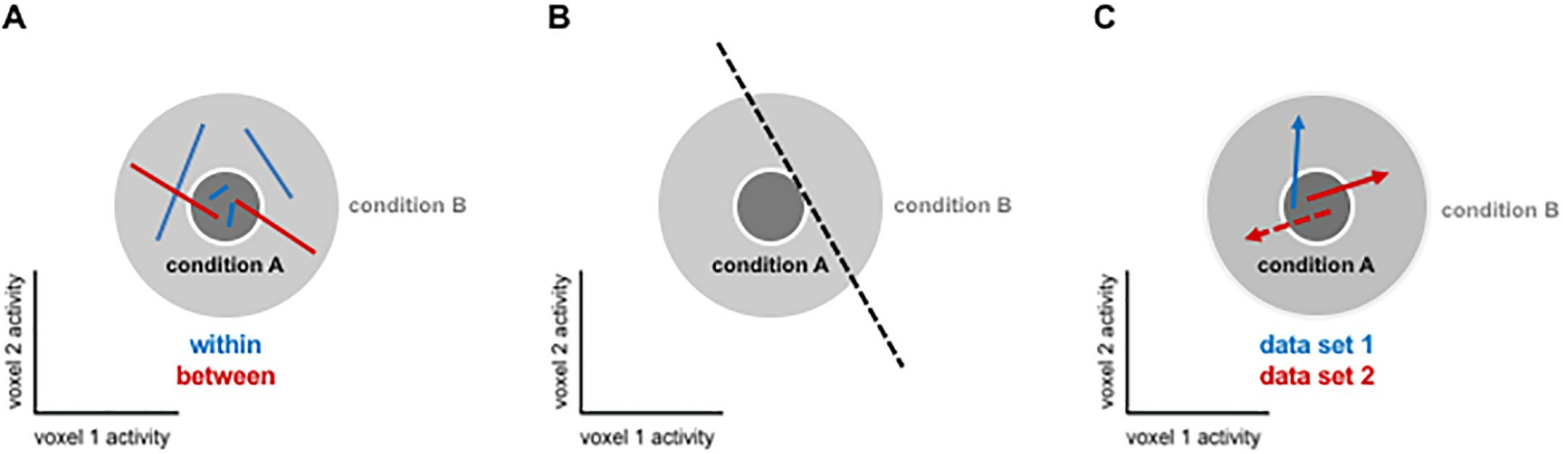
EDI and linear decoding accuracy are sensitive to differences in variance between conditions, but crossvalidated distance estimators are not. Consider two distributions of pattern estimates for two conditions (A, dark gray; B, light gray), which have identical means (central point), but different variances (small for A, large for B). (A) EDI: The mean distance between conditions (red) is larger than the mean distance within conditions (blue), because the distances within condition A are very small. The EDI, therefore, is sensitive to the difference between the conditions. (B) Linear decoder: A linear decoder may maximise its accuracy by placing the decision boundary to one side of the condition-A distribution. This way the decoder gets 100% accuracy for condition A. For condition B, accuracy is below chance (50%), but above 0. On average across conditions, the accuracy will be above chance (50%). Linear decoders, therefore, are sensitive to differences in variance (like the EDI). (C) Crossvalidated distance estimator: A crossvalidated distance estimator (e.g. crossnobis/LDC or LD-t) is a mean of inner products of pattern-difference vectors (arrows: condition-B pattern estimate minus condition-A pattern estimate). On each fold of crossvalidation, an inner product is computed between a training-set vector (blue) and a test-set vector (red). When the distributions are point-symmetric about the mean (e.g. Gaussian), each test-set vector (solid-line red arrow) has an equally probable opposite twin (dashed-line red arrow). The inner products of the training-set vector with the twin test-set vectors (red) will have opposite sign and the same absolute value. These pairs of values will cancel in the expectation. Crossvalidated distance estimates are therefore insensitive to variance differences (as long as the distributions of the pattern estimates are point-symmetric, e.g. non-isotropic Gaussian, for both conditions).

**Fig 10:**
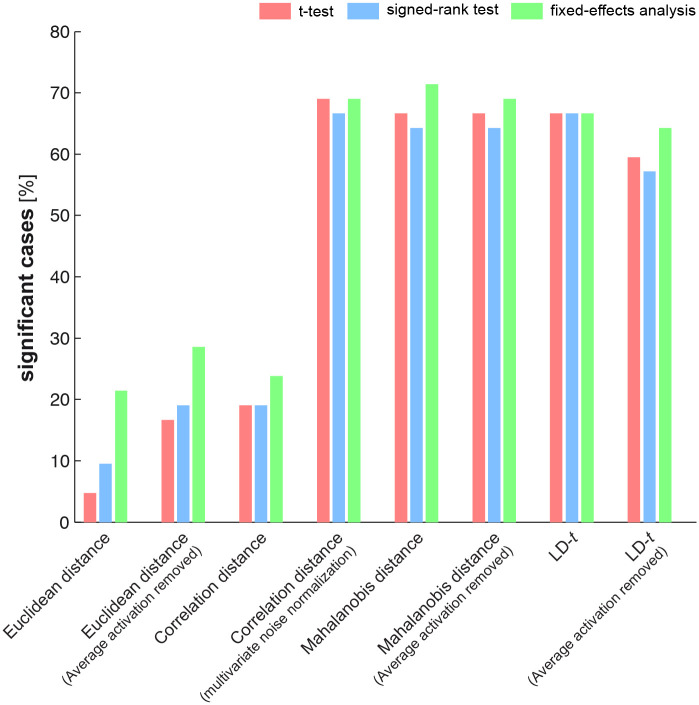
Comparing the sensitivity of different tests. Each test was applied to fMRI data from six different ROIs. In each ROI, discriminabilities of seven category subsets were assessed. A test was pronounced significant if p < 0.001. The height of the bars shows the percentage of significant cases out of the 42 tested scenarios (e.g. 50% corresponds to 21 significant tests). Tests on EDIs computed from multivariately noise-normalized response patterns were significantly more powerful (the last five triple of bars). Inference was done using subject bootstrap. All pairwise comparisons between the two groups of tests *(i*.*e*. the first three triple of bars and the next five triples of bars corresponding to unnormalized and multivariate noise-normalized data) were significant after controlling the expected false discovery rate at 0.01.
